# Methodological Issues and Evidence of Malfeasance in Research Purporting to Show Thimerosal in Vaccines Is Safe

**DOI:** 10.1155/2014/247218

**Published:** 2014-06-04

**Authors:** Brian Hooker, Janet Kern, David Geier, Boyd Haley, Lisa Sykes, Paul King, Mark Geier

**Affiliations:** ^1^Simpson University, 2211 College View Drive, Redding, CA 96001, USA; ^2^Institute of Chronic Illness, Inc., 14 Redgate Court, Silver Spring, MD 20905, USA; ^3^University of Texas Southwestern Medical Center at Dallas, Dallas, TX 75235, USA; ^4^University of Kentucky, Lexington, KY 40506, USA; ^5^CoMeD, Inc., Silver Spring, MD, USA

## Abstract

There are over 165 studies that have focused on Thimerosal, an organic-mercury (Hg) based compound, used as a preservative in many childhood vaccines, and found it to be harmful. Of these, 16 were conducted to specifically examine the effects of Thimerosal on human infants or children with reported outcomes of death; acrodynia; poisoning; allergic reaction; malformations; auto-immune reaction; Well's syndrome; developmental delay; and neurodevelopmental disorders, including tics, speech delay, language delay, attention deficit disorder, and autism. In contrast, the United States Centers for Disease Control and Prevention states that Thimerosal is safe and there is “no relationship between [T]himerosal[-]containing vaccines and autism rates in children.” This is puzzling because, in a study conducted directly by CDC epidemiologists, a 7.6-fold increased risk of autism from exposure to Thimerosal during infancy was found. The CDC's current stance that Thimerosal is safe and that there is no relationship between Thimerosal and autism is based on six specific published epidemiological studies coauthored and sponsored by the CDC. The purpose of this review is to examine these six publications and analyze possible reasons why their published outcomes are so different from the results of investigations by multiple independent research groups over the past 75+ years.

## 1. Introduction

Thimerosal is an organic-mercury (Hg) based compound, used as a preservative in many childhood vaccines, in the past and present. To date, there have been over 165 studies that focused on Thimerosal and found it to be harmful [1, 2]. (A comprehensive list of these studies is shown at http://mercury-freedrugs.org/docs/20140329_Kern_JK_ExcelFile_TM_sHarm_ReferenceList_v33.xlsx.) Of these studies, 16 were conducted to specifically examine the effects of Thimerosal on human infants and/or children [3–18]. Within these studies, which focused on human infants and/or children, the reported outcomes following Thimerosal exposure were (1) death [3]; (2) acrodynia [4]; (3) poisoning [5]; (4) allergic reaction [6]; (5) malformations [7]; (6) autoimmune reaction [8]; (7) Well's syndrome [9]; (8) developmental delay [10–13]; and (9) neurodevelopmental disorders, including tics, speech delay, language delay, attention deficit disorder, and autism [10, 11, 14–18].

However, the United States (US) Centers for Disease Control and Prevention (CDC) still insists that there is “no relationship between [T]himerosal[-]containing vaccines and autism rates in children” [19]. This is a puzzling conclusion because, in a study conducted directly by the CDC, epidemiologists assessed the risk for neurologic and renal impairment associated with past exposure to Thimerosal-containing vaccine (TCV) using automated data from the Vaccine Safety Datalink (VSD) and found a 7.6-fold increased risk of autism from exposure to Thimerosal during infancy [20]. The database for that study was “from four health maintenance organizations [HMOs] in Washington, Oregon, and California, containing immunization, medical visit, and demographic data on over 400,000 infants born between 1991 and 1997.” In that initial study, Verstraeten et al. [20] “categorized the cumulative ethyl-Hg exposure from [T]himerosal[-]containing vaccines after one month of life and assessed the subsequent risk of degenerative and developmental neurologic disorders and renal disorders before the age of six.” They “applied proportional hazard models adjusting for HMO, year of birth, and gender, and excluded premature babies.” The reported results showed that “the relative risk (RR) of developing a neurologic development disorder was 1.8 (95% confidence intervals [CI] 1.1–2.8) when comparing the highest exposure group at 1 month of age (cumulative dose > 25 *μ*g) to the unexposed group.” Similarly, they “also found an elevated risk for the following disorders: autism (RR 7.6, 95% CI = 1.8–31.5), nonorganic sleep disorders (RR 5.0, 95% CI = 1.6–15.9), and speech disorders (RR 2.1, 95% CI = 1.1–4.0)” in the highest exposure group.

Considering the many peer-reviewed published research studies that have shown harm from Thimerosal, including studies in which Thimerosal exposure is associated with the subsequent diagnosis of neurodevelopmental disorders (16 studies) such as autism, and the just-described evidence from the CDCs own research, which found evidence of a relationship between the level of Thimerosal exposure and the risk of a subsequent autism diagnosis, how does the CDC conclude that there is no evidence of that relationship? The foundation for the CDC's current stance apparently is based primarily on six specific published epidemiological studies that the CDC has completed, funded, and/or cosponsored, starting in the late 1990s. These studies include (1) the Madsen et al. [21] ecological study of autism incidence versus Thimerosal exposure in Denmark, (2) the Stehr-Green et al. [22] ecological study of autism incidence versus Thimerosal exposure in Denmark, Sweden, and California, (3) the Hviid et al. [23] study of autism incidence versus Thimerosal exposure in Denmark (also ecological), (4) the Andrews et al. [24] cohort study of autism incidence and Thimerosal exposure in the United Kingdom, (5) the published Verstraeten et al. [25] CDC cohort study of autism incidence and Thimerosal exposure in the United States, and (6) the more recent Price et al. [26] case-control study of autism incidence and Thimerosal exposure in the United States. Although the CDC cites several other publications to purport the safety of Thimerosal, only these six specifically consider its putative relationship to autism.

The purpose of this review is to examine these six publications [21–26] which were “overseen” by the CDC and which claim that prenatal and early childhood vaccine-derived Thimerosal exposures are not related to the risk of a subsequent diagnosis of autism or autism spectrum disorder (ASD). This review analyzes possible reasons why their published outcomes are so different from the results of investigations by multiple independent research groups over the past 75+ years. The review begins with an examination of the Madsen et al. [21] study.

## 2. The Madsen et al. 2003 Study

The CDC-sponsored Madsen et al. [21] study examined whether discontinuing the use of TCVs in Denmark led to a decrease in the incidence of autism. Data were obtained from the Danish Psychiatric Central Research Register, which contains all psychiatric admissions since 1971 and all outpatient contacts in psychiatric departments in Denmark since 1995. The study authors examined the data from 1971 to 2000 and reported that rate of autism increased with the removal of Thimerosal from vaccines (starting in 1992, the year that Thimerosal-containing early childhood vaccines were phased out).

Although there are several concerns about the methodology used, the most serious concern involves diagnosis. As described in the paper, estimates of total autism cases in Denmark were only based on diagnoses occurring during inpatient visits from 1971 to 1994 and then during both inpatient and outpatient visits from 1995 to the last year of the study in 2000. Thus, the inclusion criteria are greatly expanded two years after the phaseout of Thimerosal from infant vaccines in Denmark, creating an “artificial increase” in autism prevalence. The authors conceded that “the proportion of outpatient to inpatient activities was about 4 to 6 times as many outpatients as inpatients with variations across time and age bands.” However, in an earlier publication by Madsen et al. [27], the same authors had stated regarding this same data, “in our cohort, 93.1% of the children were treated only as outpatients…” Unlike the statement in the Madsen et al. [21] study, the 2002 paper indicates that the ratio between outpatients and inpatients in the 1971–2000 dataset was 13.5 : 1, which would account for an even greater increase in cases diagnosed starting in 1995 (i.e., after the probable completion of the phaseout of TCVs that started in 1992).

In addition, the authors stated that the Danish registry which was used to count cases did not include a large Copenhagen clinic before 1993. This clinic accounted for as many as 20% of the autism cases nationwide, which would again artificially inflate the autism incidence observed in Denmark after the phaseout of TCVs was initiated in 1992. The authors do not mention this change in inclusion criteria (i.e., the addition of a new clinic in the registry) neither do they attempt to adjust their analysis in accordance with the anomaly. It was revealed, instead, in a similar paper by Stehr-Green et al. [22] where the authors state regarding the Denmark registry of autistic patients, “Prior to 1992, the data in the national register did not include cases diagnosed in one large clinic in Copenhagen (which accounts for approximately 20% of cases occurring nationwide).”

Also, the diagnosis criteria for “autism” changed within the course of the study. From 1971 to 1993, the ICD-8 standards for diagnosis (psychosis protoinfantilis 299.00 or psychosis infantilis 299.01) were used to measure autism incidence. However, from 1994 to 2000, the ICD-10 standard (infantile autism, F84.1) was used. Although the authors did not address the impact of the change in diagnostic criteria, this could result in as much as a 25-fold increase in cases as the instantaneous change in autism prevalence in Denmark, due to this change, went from a low of 1.2/10,000 to a high of 30.8/10,000 [28].

Another disconcerting methodological issue was that the 2001 data, which showed a strong downward trend in autism rates in at least two of the three age groups (continuing from 1999 through 2001), was not included in the final publication. This was apparent because when the paper was initially submitted for publication, it included the 2001 data. After the paper was rejected for publication by the Journal of the American Medical Association (JAMA) and the Lancet, it was submitted to the journal Pediatrics again including the 2001 data. As stated by one of the peer-reviewers of the Pediatrics submission, “The drop of incidence shown for the most recent years is perhaps the most dramatic feature of the figure, and is seen in the oldest age group as well as the youngest. The authors do not discuss whether incomplete ascertainment in the youngest children or delay in recording of data in the most recent years might play a role in this decline, or the possibility that this decrease might have come about through elimination of [T]himerosal” (January 23, 2003, communication between Dr. Poul Thorsen, Aarhus University, and Dr. Coleen Boyle, CDC scientist). In response to this criticism, the authors removed the 2001 incidence numbers. The authors' decision to withhold these data resembles scientific malfeasance, especially when coupled with the previously discussed problematic methods for counting autism cases. If the scientists believed that downward trend between 1999 and 2001 was caused by some phenomenon unrelated to the phaseout of the TCVs, these scientists should have included those data and then explained the trend within the discussion of the data.

If the 2001 data had been included in the final publication, the results would have been consistent with a more recent CDC study [29] where a decreasing trend of autism prevalence in Denmark after the removal of Thimerosal in 1992 was reported. Instead of large increases in autism prevalence after 1992, the recent Danish study revealed that the autism spectrum disorder prevalence in Denmark fell steadily from a high of 1.5% in 1994-95 (when children receiving Thimerosal-free formulations were too young to receive an autism diagnosis and, because of the known offset in diagnosis, most of those being diagnosed had been born 4 to 8 years earlier [from 1985 to 1990]) to a low of 1.0% in 2002–2004 (more than 10 years after the phasein of the use of Thimerosal-free vaccine formulations was started in 1992).

## 3. The Stehr-Green et al. 2003 Study

The CDC's Stehr-Green et al. [22] study compared the prevalence/incidence of autism in California, Sweden, and Denmark with average exposures to TCVs. Graph-based ecologic analyses were used to examine population data from the state of California (national immunization coverage surveys and counts of children diagnosed with autism-like disorders seeking special education services in California); Sweden (national inpatient data on autism cases, national vaccination coverage levels, and information on use of all vaccines and vaccine-specific amounts of Thimerosal); and Denmark (national registry of inpatient/outpatient-diagnosed autism cases, national vaccination coverage levels, and information on use of all vaccines and vaccine-specific amounts of Thimerosal).

The study followed and appeared to be conducted in response to California study data [30], which was presented to the Institute of Medicine's Immunization Safety Review Committee. The California data showed that increased uptake of Thimerosal-containing vaccines in California during the 1990s correlated with a corresponding increase in autism diagnoses. In the Stehr-Green et al. [22] study, the researchers stated that the reliability of the autism prevalence data, citing that the California data included autism spectrum disorder diagnoses such as pervasive development disorder (PDD), could account for the increase. However, in a published response to this paper, Blaxill and Stehr-Green [31] stated that the California prevalence rates were reported based solely on autism cases.

In the Stehr-Green paper, the Sweden autism prevalence data showed an increase in autism rates from 5- 6 cases per 100,000 in 1980–82 to a peak of 9.2 cases per 100,000 in 1993. In Sweden, TCVs were phased out starting in 1987. Denmark's autism prevalence data was identical to that reported in the Madsen et al. [21] study critiqued previously. For Denmark, the authors reported an astounding 20-fold increase in autism prevalence between 1990 and 1999, despite the phaseout of TCVs that started in 1992.

In addition, the data from Sweden were based on inpatient (hospital) visits only. This limitation (counting a small fraction of the total number of cases) likely accounted for the erratic swings in the annual numbers of autism cases reported in that country. Also, the Thimerosal exposure level based on the Swedish vaccination schedule during this time period was much less (a nominal maximum of 75 *μ*g of Hg by two years of age) than that possible in California (and the United States as a whole) where developing children nominally received up to 237.5 *μ*g of Hg by 18 months of age through the standard immunization schedule. In conclusion, the Stehr-Green et al. study was problematic in its attempt to combine ecological data from three different countries that, relative to each other, demonstrated different vaccination policies and widely different Thimerosal exposure levels.

## 4. The Hviid et al. (2003) Study

The Hviid et al. [23] population-based cohort study, widely cited by the CDC, compared rates of autism prevalence among individuals who received Thimerosal-free vaccines to those receiving TCVs. The authors report that there was no evidence of increased autism prevalence with Thimerosal exposure.

The study authors stated that the mean age of autism diagnosis within their population was 4.7 years with a standard deviation of 1.7 years. However, cases and controls as young as 1 year of age were included within the analysis. Accordingly, controls that were less than the mean age of diagnosis minus two standard deviations (1.3 years) from that age had a 97.5% probability of actually being individuals who will later develop autism and are therefore possibly misclassified. Similarly, in this study, the mean age for an ASD diagnosis was 6.0 years with a standard deviation of 1.9 years. Thus, the study methodology is questionable because it appears to have underascertained the number of cases diagnosed with autism and ASD.

In addition, rather than counting persons within the cohort, the authors counted “person-years of follow up.” With this technique, each age group (one-year-olds, two-year-olds, etc.) was considered equally, despite the fact that younger age groups were much less likely to receive an autism diagnosis. This again contributed to the undercounting of the cases with a diagnosis of autism and ASD and biased the study towards the null hypothesis (that there is no statistically significant Thimerosal exposure effect on the outcomes observed).

## 5. The Andrews et al. (2004) Study

The Andrews et al. [24] study was a retrospective cohort study completed using records from a database in the United Kingdom, where autism prevalence rates were compared for children receiving Thimerosal-containing DTaP and DT vaccines. In the Andrews et al. [24] study, Cox's proportional-hazards ratios were used to evaluate periods of followup in the cohort examined by the investigators using the records in the general practitioner research database (GPRD), a database that was known to have a significant level of errors. These investigators reported that increased organic-Hg exposure from TCVs was associated with a significantly reduced risk for diagnosed general developmental disorders and for unspecified developmental delay (although there was a significantly higher risk for diagnosed tics).

Considering that there are several studies conducted by independent investigators that have found that exposure to Thimerosal is a risk factor for neurodevelopmental delay and disorders [10, 11, 16], the reduced rate of neurodevelopmental delay and disorders with Thimerosal exposure found in the Andrews et al. [24] study suggests possible methodological issues.

This result may have occurred, in part, because other studies examined cohorts with significantly different childhood vaccine schedules and with different diagnostic criteria for outcomes. This difference may also exist because these other studies that found Thimerosal to be a risk factor for neurodevelopmental delay and disorders employed different epidemiological methods, especially with respect to the issue of follow-up period for individuals in the cohorts examined. The method used to measure follow-up period for individuals is a critical issue in all studies examining the relationship between exposures and the subsequent risk of a neurodevelopmental disorder diagnosis, especially in those instances where the postexposure periods for all of the participants in the study are essentially the same. This is because the risk of an individual being diagnosed with a neurodevelopmental disorder is not uniform throughout his/her lifetime. As observed in the present study, the initial mean age for any neurodevelopmental disorder diagnosis was 2.62 years old, and the standard deviation of mean age of the initial diagnosis of neurodevelopmental disorder was 1.58 years old. These findings are highly problematic because (1) any follow-up method that fails to consider the lag time between birth and age of initial neurodevelopmental disorder diagnosis will likely not be able to observe the true relationship between exposure and the subsequent risk of a neurodevelopmental disorder diagnosis and (2) statistically, the mean and standard deviation age of diagnosis as reported lead to the nonsensical result that a significant portion (2.5%) of the children in this study were diagosed with a neurodevelopmental disorder more than six months before they were born (i.e., the mean age minus two standard deviations, 2.62 − [2 × 1.58] = −0.54 years of age).

Another issue with this study is that the authors used a nontransparent, multivariate regression technique to analyze vaccine uptake and autism prevalence data. The study included one dependent variable (autism) and multiple independent variables, including two independent variables (Thimerosal exposure levels and year of birth) that were “correlated” with each other, since Thimerosal exposures increased with time. Thus, the researchers did not report a statistical analysis of the effect of Thimerosal exposure on autism incidence, despite the fact that the authors stated that no such effect was observed. Moreover, the methods used in this study can create a problem in regression known as “multicolinearity.” In this case, since the time variable and the vaccine exposure variable are correlated, they actually compete to explain the outcome effect. Inclusion of the time variable reduces the significance of the exposure variable. Yet, the authors did not explain why they included a time variable that competes with the exposure variable. Unfortunately, the authors of this study never released the raw data so that a valid single-variable analysis could be conducted to ascertain the probability of an association between Thimerosal exposure and the risk of autism.

It is also important to note that the UK Thimerosal exposure (a maximum of 75 *μ*g of Hg by 4 months of age) was not comparable to that in the United States (a maximum of 75 *μ*g of Hg by 2 months of age and 187.5 *μ*g of Hg by 6 months of age). Thus, this study should not be extrapolated to the probability of an autism-Thimerosal association based on the US vaccination schedule.

## 6. The Verstraeten et al. (2003) Study

The CDC's published Verstraeten et al. [25] study consists of a cohort analysis of a subset of records from the medical records databases for several of the HMOs whose records were maintained in a central data repository, the Vaccine Safety Datalink (VSD). This study was conducted in at least five separate phases. In the final phase (i.e., the results reported in the publication), the authors stated that there was no relationship between Thimerosal exposure in vaccines and autism incidence. However, no data are reported in the published study to support this conclusion.

Results from the first phase of the study released in an internal presentation abstract by Verstraeten et al. [20] (mentioned earlier) using records from four (4) HMOs showed that infants who were exposed to greater than 25 *μ*g of Hg in vaccines and immunoglobulins at the age of one month were 7.6 times more likely to have an autism diagnosis than those not exposed to any vaccine-derived organic Hg. Within the same abstract, Verstraeten reports that the risk for any neurodevelopmental disorder was 1.8, the risk for speech disorder was 2.1, and the risk for nonorganic sleep disorder was 5.0. All relative risks were statistically significant.

In the second phase of the study, a different approach was taken: exposure was compared at 3 months of age, rather than one month. Results of this phase showed that children exposed to the maximum amount of organic Hg in infant vaccines (62.5 *μ*g) were 2.48 times more likely to have autism diagnosis compared to those exposed to less than 37.5 *μ*g of Hg in vaccines. These results were also statistically significant. No assessment against a “no exposure” control was apparently completed in this study phase.

**Figure 1:**
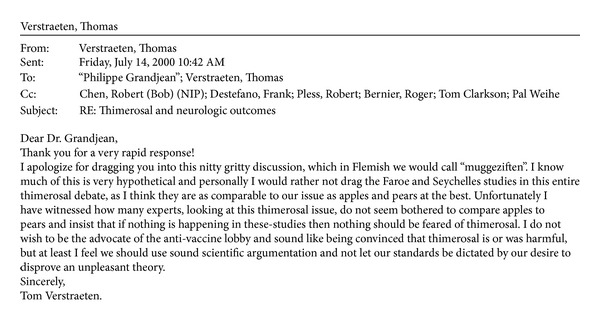
July 14, 2000, email from Verstraeten to Philippe Grandjean regarding the risk of harm due to Thimerosal (obtained by the authors via the US Freedom of Information Act of 1950 as amended).

In the third phase of the study, in which more data stratification methods and different inclusion/exclusion criteria were applied to the analysis, the relative risk of autism for children at three months of Thimerosal exposure dropped to 1.69. At this point, evidence in an email from Verstraeten, the lead investigator, written to a colleague outside of the CDC (obtained by the authors via the US Freedom of Information Act of 1950 as amended), suggests that Verstraeten could have been receiving pressure within the CDC to apply unsound statistical methods to deny a causal relationship between Thimerosal and autism. In this email, Verstraeten states (Figure 1), “I do not wish to be the advocate of the anti-vaccine lobby and sound like being convinced that thimerosal is or was harmful, but at least I feel we should use sound scientific argumentation and not let our standards be dictated by our desire to disprove an unpleasant theory.”

The fourth and fifth phase of the study used records from only two of the original HMOs and incorporated a third HMO, Harvard Pilgrim, into the analysis. Some critics of the study questioned the use of Harvard Pilgrim, as this HMO appeared to be riddled with uncertain record keeping practices, and the state of Massachusetts had been forced to take it over after it declared bankruptcy. In addition, the HMO used different diagnostic codes than the other two HMOs used in phases 2 and 3. Other criticisms include that the study used younger children, from 0 to 3 years of age, even though the average age for an autism diagnosis at the time was 4.4 years. Since half of the children receiving an autism diagnosis would be over 4.4 years of age, far greater than the maximum age in the study at 3 years, this analysis excluded more than 50% of all autism cases from this HMO. Also, the cohort from this HMO contained 7 times fewer individuals than the main cohort from the previous study (i.e., HMO B), and there was no apparent attempt to assess the power of this HMO to show any statistically significant effect.

Also of note is the lack of variability within strata among the different HMOs in the Verstraeten et al. [25] study. By design, a cohort study seeking to assess the effect of some treatment on a subsequent outcome should be designed to maximize the range of the independent “treatment” variable (Thimerosal exposure in this instance) in order to determine if there is indeed an “effect” in the dependent postexposure outcome variable (neurological disorders in this study). However, the authors knowingly stratified the analysis based on the participants' gender, year of birth, month of birth, and clinic most often visited. This effectively reduced the variability of Thimerosal exposure within the strata to the point that it reduced the capability of the final analysis to find any but the “strongest” Thimerosal exposure-related outcome effects. The problems with such “overmatching” practices have been discussed in detail in peer-reviewed scientific literature and will be treated in greater detail in the forthcoming review of the CDC's Price et al. [26] paper.

Another methodological concern about the Verstraeten et al. [25] study is related to the issue of the minimum follow-up period required for individuals in the cohorts examined to ensure that all the cases in the cohort will have been identified with a high degree of certainty. This issue has been mentioned as a problem in the previous studies. As mentioned earlier, the method used to determine the minimum follow-up period for individuals is a critical issue in all studies examining the relationship between exposures and the subsequent risk of a neurodevelopmental disorder diagnosis, especially in those instances where the exposures to all participants in the study are the same or essentially the same. This is the case because the risk of an individual being diagnosed with a neurodevelopmental disorder is not uniform throughout his/her lifetime. Any follow-up method that fails to consider the lag time between birth and age of initial neurodevelopmental disorder diagnosis will likely not be able to observe the true relationship between exposure and the subsequent risk of a neurodevelopmental disorder diagnosis. Verstraeten et al. [25] included children in the control group who were too young (down to “0” years of age) to receive a neurodevelopmental disorder diagnosis.

Within this study, Verstraeten et al. [25] still found significantly increased risk ratios for tics and language delay. However, the authors stated that, because these results were not consistent between the HMOs tested, these significantly increased risk ratios could not be used to make a determination of the potential adverse consequences of organic-Hg exposure from TCVs.

## 7. The Price et al. 2010 Study

In 2010, the CDC published another epidemiology study on Thimerosal and autism [26]. This case-control study was conducted using the records from three managed care organizations (MCOs) consisting of 256 children with an ASD diagnosis and 752 controls that were matched by birth year, gender, and MCO to the children with an ASD diagnosis. Exposure to Thimerosal in vaccines and immunoglobulin preparations was determined from electronic immunization registries, medical charts, and parent interviews. Conditional logistic regression was used to assess associations between ASD, autistic disorder (AD), and ASD with regression and exposure to ethyl-Hg during prenatal, birth-to-1-month, birth-to-7-month, and birth-to-20-month periods. Their published finding was that prenatal and infant Thimerosal exposure from TCVs and Thimerosal-containing immunoglobulin posed no statistically significant risk of autism.

As mentioned earlier, in case-control studies, the main methodological concern is the phenomenon called “overmatching.” This concern for overmatching in the Price et al. [26] study was voiced previously by DeSoto and Hitlan [32]. In their comprehensive analysis of overmatching errors specific to the Price paper, DeSoto and Hitlan [32] stated that “Matching cannot—or should not—be done in a way that artificially increases the chance that within[-] strata exposure is the same; this happens when a matching variable is a significant predictor of exposure and is called overmatching.”

Cases were matched with controls of the same age and sex, within the same HMO and essentially the same vaccination schedule, using the same vaccine manufacturers. DeSoto and Hitlan then state further, regarding the lack of variability of Thimerosal exposure in the Price study, “Across the different years, the average cumulative exposure varies from 42.3 *μ*g to 125.46 *μ*g; while within the birth year stratas (sic), the mean exposures do not vary by more than 15 micrograms.” In other words, the maximum level of variation in Thimerosal exposure in the cases and controls being compared was 15 *μ*g of Hg, as compared to the “83” *μ*g of Hg range for the average cumulative exposures in the cohort studies. Moreover, this range is much less than the range of Thimerosal exposures that could have been used to determine risk including (a) 0 to 50 *μ*g of Hg for one-month exposures, (b) 0 to 190 *μ*g of Hg for seven-month exposures, and (c) 0 to 300 *μ*g of Hg for 20-month exposures. Finally, regarding the Price study, DeSoto and Hitlan [32] concluded, “this paper is flawed. Unfortunately, there is not an analytic fix for overmatching: it is [a] design flaw.”

Prenatal Thimerosal exposure for the children within the study arose from the Thimerosal-preserved inactivated-influenza vaccine given during pregnancy and the Rho immunoglobulin administered to pregnant women to prevent Rh-factor incompatibility injury to the developing child. Unlike postnatal exposure from TCVs in the recommended childhood vaccination schedule, prenatal exposures would not be overmatched in a study design that stratified the participants based on their birth year or HMO. Evidence from the background CDC report regarding the Price study showed a significant risk of regressive autism due to prenatal Thimerosal exposure levels, at exposure levels as low as 16 *μ*g of Hg [33]. However, the risk of regressive autism due to prenatal Thimerosal exposure reported in that paper was 1.86 and yielded a *P* value of 0.072 which was deemed as insignificant based on the authors' “cut-off” value of *P* < 0.05. However, *P* values between 0.05 and 0.10 are “marginally significant” and should merit further study. In addition, upon further analysis, it was found that the 2009 background report [33] to the Price et al. [26] study showed that the prenatal Thimerosal exposure model was run in six different ways and that the most reliable methods (those that factored out the postnatal Thimerosal exposure effects) found highly statistically significant relative risks of up to 8.73 (*P* = 0.009) for regressive ASD due to prenatal Thimerosal exposures from Thimerosal-containing influenza vaccines and Rho immunoglobulin products relative to no such prenatal Thimerosal exposures. Curiously, these more compelling results were not reported in the paper. Withholding these data from the publication and, instead, reporting a significantly lower value could appear to constitute scientific malfeasance on the part of the authors of this study.

## 8. Conclusion

As seen in this review, the studies upon which the CDC relies and over which it exerted some level of control report that there is no increased risk of autism from exposure to organic Hg in vaccines, and some of these studies even reported that exposure to Thimerosal appeared to decrease the risk of autism. These six studies are in sharp contrast to research conducted by independent researchers over the past 75+ years that have consistently found Thimerosal to be harmful. As mentioned in the Introduction section, many studies conducted by independent investigators have found Thimerosal to be associated with neurodevelopmental disorders. Several studies, for example, including three of the six studies covered in this review, have found Thimerosal to be a risk factor for tics [10, 17, 24, 25, 34, 35]. In addition, Thimerosal has been found to be a risk factor in speech delay, language delay, attention deficit disorder, and autism [10, 11, 15–17, 24, 25, 34].

Considering that there are many studies conducted by independent researchers which show a relationship between Thimerosal and neurodevelopmental disorders, the results of the six studies examined in this review, particularly those showing the protective effects of Thimerosal, should bring into question the validity of the methodology used in the studies. A list of the most common methodological issues with these six studies is shown in Table 1. Importantly, other than the Hviid et al. [23] study, five of the publications examined in this review were directly commissioned by the CDC, raising the possible issue of conflict of interests or research bias, since vaccine promotion is a central mission of the CDC. Conceivably, if serious neurological disorders are found to be related to Thimerosal in vaccines, such findings could possibly be viewed as damaging to the vaccine program.

## Figures and Tables

**Table 1:** Methodological issues most common in each of the six reviewed studies.

Study reviewed	Methodological issues
Madsen et al. [21]	(i) Changing entrance criteria in ecological studies. (ii) Withholding important results from the final publication. (iii) Conclusions not generalizable to the US vaccination schedule due to widely different vaccination schedules and different levels of Thimerosal dosing in other countries.

Stehr-Green et al. [22]	(i) Changing entrance criteria in ecological studies. (ii) Withholding important results from the final publication. (iii) Conclusions not generalizable to the US vaccination schedule due to widely different vaccination schedules and different levels of Thimerosal dosing in other countries.

Hviid et al. [23]	(i) Accounting for “person-years” regarding exposure rather than actual exposure levels. (ii) Conclusions not generalizable to the US vaccination schedule due to widely different vaccination schedules and different levels of Thimerosal dosing in other countries.

Andrews et al. [24]	(i) Accounting for “person-years” regarding exposure rather than actual exposure levels. (ii) Conclusions not generalizable to the US vaccination schedule due to widely different vaccination schedules and different levels of Thimerosal dosing in other countries.

Verstraeten et al. [25]	(i) Cohort of children too young for followup for an autism diagnosis. (ii) “Overmatching” phenomena due to too closely matched cases and controls. (iii) Withholding important results from the final publication.

Price et al. [26]	(i) “Overmatching” phenomena due to too closely matched cases and controls. (ii) Withholding important results from the final publication.
